# Lactoferrin translocates to the nucleus of bovine rectal epithelial cells in the presence of *Escherichia coli* O157:H7

**DOI:** 10.1186/s13567-019-0694-3

**Published:** 2019-10-01

**Authors:** Joanna Rybarczyk, Dmitry Khalenkow, Evelien Kieckens, Andre G. Skirtach, Eric Cox, Daisy Vanrompay

**Affiliations:** 1 Department of Animal Sciences and Aquatic Ecology, Faculty of Bioscience Engineering, Ghent University, 9000 Ghent, Belgium; 2 Department of Molecular Biotechnology, Faculty of Bioscience Engineering, Ghent University, 9000 Ghent, Belgium; 3 Laboratory of Immunology, Faculty of Veterinary Medicine, Ghent University, 9000 Ghent, Belgium

## Abstract

Enterohemorrhagic *Escherichia coli* (EHEC) O157:H7 is a foodborne pathogen which causes illness in humans. Ruminants are the main reservoirs and EHEC predominantly colonizes the epithelium of the recto-anal junction of cattle. Immunosuppression by EHEC promotes re-infection of cattle. However, bovine lactoferrin (bLF) apparently can overrule the immunosuppression by inducing EHEC-specific IgA responses at the mucosal site. The IgA responses are significantly correlated with reduced EHEC shedding and the absence of colonization at the rectal mucosa following re-infection. Therefore, to examine the interaction between bLF and bovine rectal epithelial cells, we first developed a method to establish a primary cell culture of epithelial cells of the rectum of cattle. Furthermore, we used LC–MS/MS to demonstrate the presence of secreted lactoferrin in bovine milk and the absence of a “delta” isoform which is known to translocate to the nucleus of cells. Nevertheless, lactoferrin derived from bovine milk was internalized by rectal epithelial cells and translocated to the nuclei. Moreover, nuclear translocation of bLF was significantly enhanced when the epithelial cells were inoculated with EHEC, as demonstrated by confocal fluorescence microscopy and confirmed by Raman microscopy and 3D imaging.

## Introduction

Enterohemorrhagic *Escherichia coli* (EHEC) O157:H7 is a foodborne pathogen which colonizes the colon of humans and causes illness ranging from watery or bloody diarrhea and haemorrhagic colitis to acute renal failure and haemolytic uremic syndrome (HUS) [1]. Infection in humans is mostly acquired through the ingestion of EHEC contaminated food or water, but it can also occur through direct contact with infected animals, or person-to-person transmission. Ruminants, especially cattle, are the main reservoirs for *E. coli* O157:H7, which in contrast to humans harbour the bacteria in the gastrointestinal tract without showing illness. *E. coli* O157:H7 predominantly colonizes the epithelium of the recto-anal junction of cattle, located above the gut-associated lymphoid tissue [2, 3]. Mechanisms leading to persistence of *E. coli* O157:H7 in cattle are largely unknown. However, Kieckens et al. [4] analysed the transcriptome profiles (RNA-Seq) of samples of the ileal Peyers’ patches and the recto-anal junction of calves experimentally infected with EHEC. They demonstrated upregulation of immune suppressive effects and downregulation of immunostimulatory effects on different levels of the innate and adaptive immune response. Immunosuppression promoted experimental re-infection of calves.

A number of approaches have been evaluated to prevent EHEC colonization and shedding by ruminants in order to diminish the risk of human infections [5]. So far, there is no strategy to completely protect against EHEC colonization in cattle. However, during a previous study, we demonstrated that rectal administration of bovine lactoferrin (bLF) derived from milk cleared EHEC infections at the rectal mucosa of cattle. In addition, we showed that bLF activated the mucosal immune system and induced protection against EHEC re-infection [6]. Rectal administration of bLF induced EspA- and EspB- specific mucosal IgA titers. EspA and EspB are part of the type III secretion system (TTSS) of EHEC. EspA is a major part of a filamentous needle-like structure through which TTSS effector proteins, such as EpsB, EspD and Tir, are delivered to the host cell. EspB forms pores in the host cell membrane and is also translocated into the host cell cytosol, where it triggers signal transduction events that mediate effacement of the microvilli and replacement with a pedestal-like structure. EspA and EspB-specific IgA responses at the mucosal site significantly correlated with reduced EHEC shedding and the absence of bacterial colonization at the rectal mucosa following re-infection. Thus, administration of bLF derived from milk apparently “overruled” the immunosuppression caused by EHEC. The mechanism behind the immunostimulation by bLF remains unknown. However, the specific IgA response was not detectable in the serum indicating the local nature of the protective response induced by bLF.

Lactoferrin is a conserved iron-binding glycoprotein with antimicrobial and immunomodulatory activities [7–9]. Human lactoferrin exists as different variants due to a gene polymorphisms, post-transcriptional and post-translational modifications. The two main isoforms are secreted: lactoferrin (LF) (80 kDa) [10], which is also present in animals, and its nucleocytoplasmic counterpart, delta-lactoferrin (∆LF) (73 kDa) [11, 12], which is as far as we know, not (yet) found in animals. Human LF and ∆LF are derived from the transcription of the gene encoding LF at alternative promoters. LF is present in the secondary granules of neutrophils, in serum and it is secreted by epithelial cells into exocrine fluids of mammals like colostrum, milk, tears, saliva, vaginal fluids, gastrointestinal fluids, bile, urine, and sweat [10, 13–16]. ∆LF, on the other hand is the intracellular protein in which the leader sequence and the first 25 amino acid residues of LF are absent. Human ∆LF clearly translocates to the nucleus and acts as a nuclear transcription factor [11, 17–21], whereas the nuclear targeting of human or bovine LF is still controversial [11, 19, 22–24].

Bovine rectal epithelial cell lines are non-existent and as far as we know, studies on cellular localization of milk bLF have not yet been performed in bovine cells. Therefore, we developed an in vitro model consisting out of primary bovine rectal epithelial cells. Before examining the interaction of bLF with these cells, we first examined the presence of bLF isoforms in milk. Next, we studied the uptake and intracellular localization of milk bLF using confocal fluorescence microscopy, Raman microscopy and 3D-imaging. Uptake and intracellular localization of bLF in rectal epithelial cells was studied in the presence and absence of *E. coli* O157:H7.

## Materials and methods

### Isolation and characterization of bovine rectal epithelial cells

Cattle (18–24 months old) processed in an abattoir in East-Flanders (Flanders Meat Group, Zele, Belgium) were sampled at slaughter. After evisceration, feces were manually removed from the cattle rectum and specimens (approximately 10 cm^2^) of the terminal rectum (10–15 cm proximal to the anus) were collected from different animals. Rectal tissues were immediately rinsed with ice cold (4 °C) phosphate buffered saline (PBS) supplemented with 100 U/mL penicillin (Sigma, Aldrich, St. Louis, MO, USA), 100 µg/mL streptomycin (Sigma) and 25 µg/mL gentamicin (Gibco) and were held in ice-cold (4 °C) Hanks’ balanced salt solution (HBSS) (Thermo Fisher Scientific, Erembodegem, Belgium) supplemented with 100 U/mL penicillin (Sigma), 100 µg/mL streptomycin (Sigma), 25 µg/mL gentamicin (Gibco) and 2.5 µg/mL amphotericin B (Sigma) for transport to the laboratory.

Samples were immediately processed upon arrival in the laboratory. Bovine rectal crypts were isolated by enzymatic digestion in combination with mechanical disintegration as described by others with some modifications [25–28]. Briefly, the epithelium of the lamina mucosa was separated from the lamina propria by scrapping with a sterile glass slide, where after the epithelial layer was finely homogenized using a sterile surgical blade. The homogenized tissue was subsequently washed (5 min, 130 × *g*, 4 °C) three times in HBSS (4 °C) in order to remove the mucus layer. Rectal crypts were obtained by enzymatic digestion (60 min, 100 rpm, 37 °C) carried out in Dulbecco’s Modified Eagle medium (DMEM) (Gibco, Grand Island, NY) supplemented with 1% (v/v) heat inactivated fetal calf serum (FCS), 100 U/mL penicillin (Sigma), 100 µg/mL streptomycin (Sigma), 25 µg/mL gentamicin (Gibco), 2.5 µg/mL amphotericin B (Sigma) and 100 U/mL collagenase (Sigma) until isolated crypts were observed under a microscope. The crypts suspension was further disintegrated by passages through a sterile 20G × 2″ (0.9 × 51 mm) needle. Subsequently, bacteria and single cells, such as fibroblasts were removed by 5 differential centrifugation steps (5 min, 50 × *g*, 4 °C) with DMEM containing 2% sorbitol (w/v) (Sigma). Finally, the resulting crypt pellets were washed with HBSS (3 min, 65 × *g*, 4 °C) containing 100 U/mL penicillin (Sigma), 100 µg/mL streptomycin (Sigma), 2.5 µg/mL amphotericin B (Sigma) and 25 µg/mL gentamicin (Gibco).

Isolated crypts were seeded at a density of 200–250 crypts per well into 24-well tissue culture plates (Corning^®^ Costar^®^). Each well contained a rat tail collagen (5 µg/cm^2^) (Roche Diagnotics, Mannheim, Germany) pre-coated glass coverslip. Tissue culture plates were incubated at 37 °C, and 5% CO_2_ in DMEM supplemented with 100 U/mL penicillin (Sigma), 100 µg/mL streptomycin (Sigma), 25 µg/mL gentamicin (Gibco), 2.5 µg/mL amphotericin B (Sigma), 10 ng/mL epidermal growth factor (Sigma) and 25 U/mL bovine insulin (Sigma). During the first 24 h, 10% heat inactivated FCS was supplemented to the medium in order to enhance the adherence of the crypts to the polystyrene tissue culture plates. Afterwards, 1% heat inactivated FCS was used in order to reduce fibroblast migration and subsequent proliferation. Fibroblast growth was further inhibited by repeated gentle trypsinization (0.025% Trypsin–EDTA for 2–3 min at 37 °C) every 2 days. Confluence of the cultured cells was reached within 6 to 7 days and contained approximately 1.8 × 10^5^ cells/well.

The epithelial nature of the cultures grown on the glass coverslips was confirmed by cytokeratin immunofluorescence staining using the anti-pan cytokeratin mouse monoclonal antibody (isotype IgG1, clone C11; C2931; Sigma) followed by incubation with FITC-conjugated goat-anti-mouse IgG (1:80) (ThermoFisher) as described by Hoey et al. [29]. A monoclonal anti-vimentin antibody (isotype IgG1, clone V9) (V6389; Sigma) in combination with FITC-conjugated goat-anti-mouse IgG (1:80) (ThermoFisher) was used to examine the presence of fibroblasts. A final rinsing with biDest was followed by mounting of the coverslips using Mowiol (Calbiochem, VWR, Haasrode, Belgium) mounting medium. Images were acquired using a Leica TCS SP2 confocal laser scanning microscope (Leica Microsystems GmbH, 40×/NA 1.2–0.75).

### Proteomic analysis of bLF derived from milk

Bovine lactoferrin (bLF), purified (ion-exchange chromatography) from milk (iron saturation of 16% and purity of 92%) was purchased from Ingredia Nutritional (France). The presence of bLF isoforms was examined by 2D polyacrylamide gel electrophoresis (PAGE) and nanoLC–MS/MS. Proteins that were separated by 2D-PAGE and stained by Coomassie dye were excised, washed and the proteins from the gel were digested according to published protocols [30, 31]. The peptide mixture was analyzed by reversed phase nanoliquid chromatography (LC) and mass spectrometry (MS) (LC–MS/MS) using a NanoAcquity UPLC (Micromass/Waters, Milford, MA, USA) coupled to a Q-TOF Xevo G2 mass spectrometer (Micromass/Waters), according to published procedures [30, 32, 33]. Briefly, the peptides were loaded onto a 100 μm × 10 mm NanoAquity BEH130 C18 1.7 µm UPLC (Waters, Milford, MA, USA) and eluted over a 60 min gradient (short gradient) of 2–80% organic solvent (ACN containing 0.1% FA) at a flow rate of 400 nL/min and over a 120 min gradient (long gradient) of 10–85% organic solvent at a flow rate of 250 nL/min. The raw data were processed using ProteinLynx Global Server (PLGS, version 2.4) software as previously described [33]. The following parameters were used: background subtraction of polynomial order 5 adaptive with a threshold of 30%, double smoothing with a window of three channels in Savitzky-Golay mode and centroid calculation of top 80% of peaks based on a minimum peak width of 4 channels at half height. The resulting pkl files were submitted for database search and protein identification to the Mascot server (Matrix Science, London, UK) for database search using the following parameters: databases from NCBI (Mammalia), parent mass error of 0.5 Da with 1 ^13^C, product ion error of 0.8 Da, enzyme used: trypsin, three missed cleavages, propionamide as cysteine fixed modification and methionine oxidized as variable modification. To identify the false negative results, we used additional parameters such as different databases or organisms, a narrower error window for the parent mass error (1.2 and then 0.2 Da) and for the product ion error (0.6 Da), and up to two missed cleavage sites for trypsin. The Mascot database search provided a list of proteins for each gel band. To eliminate false positive results, for the proteins identified by either one peptide or a mascot score lower than 25, we verified the MS/MS spectra that led to identification of a protein.

### Preparation of fluorescent labelled bLF

For microscopy, Alexa Fluor 488 labelled bLF was prepared by use of the Alexa Fluor^®^ 488 Protein Labeling Kit according to the manufacturer’s instructions (Molecular Probes, Eugene, OR, USA).

### *Escherichia coli* O157:H7

The nalidixic acid-resistant *E. coli* O157:H7 (EHEC) strain NCTC 12 900, a well-characterized Shiga-toxin negative strain of human origin [34], was used to test the uptake and intracellular localization of bLF by rectal epithelial cells in the presence and absence of EHEC. Bacteria were grown overnight at 37 °C in 10 mL Luria–Bertani broth (LB) (Becton–Dickinson, Claix, France) while shaking (200 rpm), harvested by centrifugation (4000 × *g*, 10 min, 4 °C) and re-suspended in DMEM to a concentration of 10^7^ CFU/mL.

### Influence of EHEC O157:H7 on the intracellular localization of bLF

Localization of bLF in primary rectal epithelial cells in the presence or absence of EHEC O157:H7 was studied by confocal fluorescence microscopy, Raman microscopy and 3D-imaging. Rectal epithelial cells were grown on glass coverslips or on CaF_2_ Raman grade 75 × 25, 1 mm polished slides (Crystran Ltd., UK) for fluorescence microscopy or Raman microscopy, respectively. Obtained monolayers (6–7 days old) were washed with PBS (RT) and subsequently inoculated with 10^7^ CFU/mL of *E. coli* O157:H7 diluted in DMEM. Cultures were incubated for 5 h at 37 °C to allow cellular attachment of EHEC. Next, monolayers were washed with PBS (RT) and incubated for 2 h (37 °C, 5% CO_2_) with 100 µL (1 mg/mL DMEM) of Alexa Fluor 488-labelled bLF, non-labelled bLF or Alexa Fluor 488 dye alone. All incubations were performed in triplicate wells for each condition, with at least two biological replications. Afterwards, monolayers were washed with PBS (4 °C) and fixed with 4% paraformaldehyde (30 min, RT) for either fluorescence or Raman microscopy. For fluorescence microscopy, nuclei were counterstained using the Hoechst 33 258 nucleic acid stain (10 min, RT) (Sigma). Fluorescence images of monolayers stained with Alexa Fluor 488-labelled bLF, non-labelled bLF or Alexa Fluor 488 dye alone were acquired using a Nikon Ti confocal laser scanning microscope (Nikon, Belux, Brussels, Belgium) equipped with a Nikon Plan Apo VC 60× Oil DIC N2 objective (1.40 NA). Hoechst 33 258 nucleic acid stain positive cells (DAPI filter) and cells positive for bLF-Alexa Fluor 488 were counted using the “Cell counter” plugin from the ImageJ v.150b software.

Cells positive for bLF-Alexa Fluor 488 were mapped with an Alpha300 R confocal Raman imaging system (WITec, Ulm, Germany) to confirm the intracellular localization of bLF. The spectrum of pure bLF in PBS (35 mg/mL) was used for this purpose. Cells incubated with Alexa Fluor 488 dye were used as negative control. The confocal Raman imaging system was equipped with a with 785 nm excitation diode laser (Toptica Photonics AG, Munich, Germany) and an UHTS 300 spectrometer with a −60 °C cooled CCD camera (ANDOR iDus 401 BR-DD, Belfast, UK). An 100×/0.9 NA Nikon objective (Nikon Belux, Brussels, Belgium) was used. Spectra were acquired at each pixel of the scanning area with an integration time of 2 s and a laser power of 120mW (measured before the objective). The lateral resolution was 0.5 µm per pixel.

Bovine LF positive cells identified by Raman microscopy were used for 3D imaging by scanning the rectal epithelial cells in different Z planes with 1 µm distance between the confocal planes. Nikon Nis Elements v.4.51 software (Nikon Belux, Brussels, Belgium) was used for 3D image calculations from the stack of resulted sample slices.

### Statistical analysis

Statistical analysis was performed using GraphPad Prism 6 software. The numbers of bLF positive cells in the presence or absence of *E. coli* O157:H7 were compared using the non-parametric Mann–Whitney U test. A value of *p* < 0.05 was considered statistically significant.

## Results

### Isolation and characterization of bovine rectal epithelial cells

**Figure 1:**
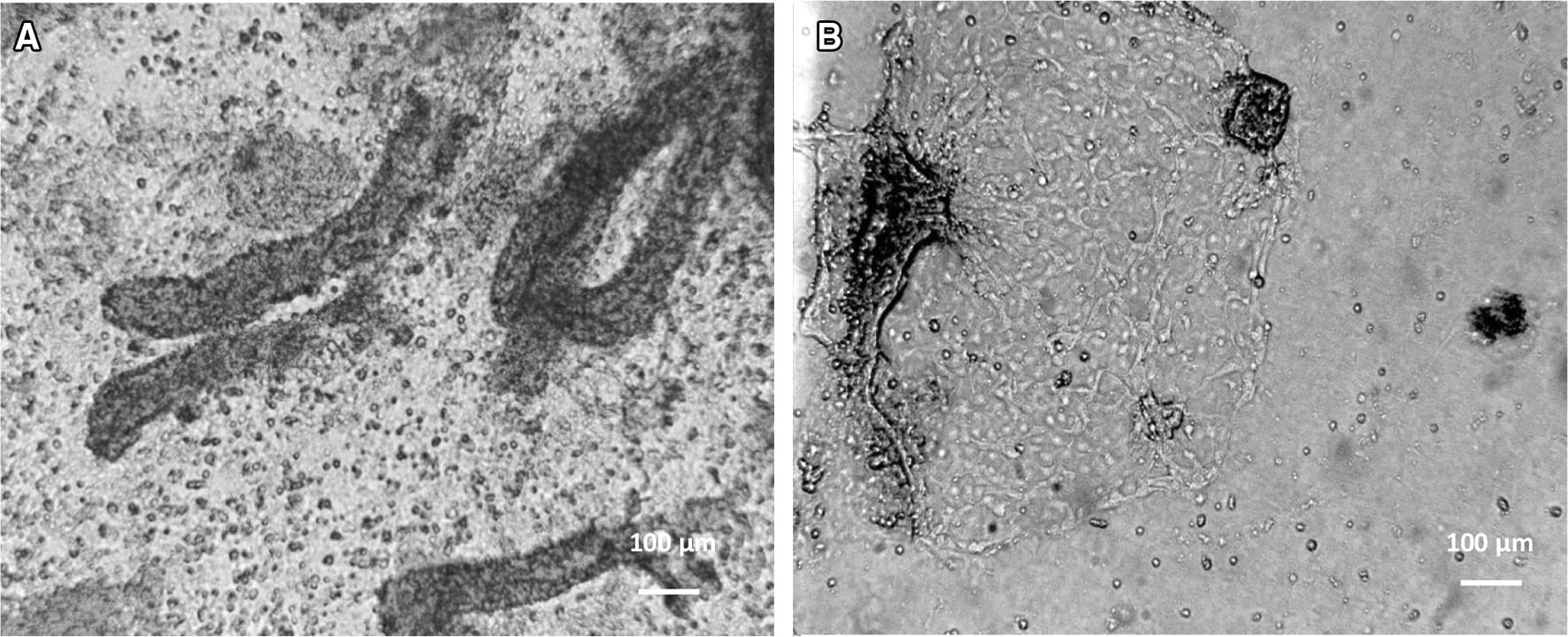
**Isolation of bovine rectal epithelial cells.** The light microscopic images of: **A** crypts of the rectal mucosa after 1 h of enzymatic digestion with collagenase (×10), **B** island of cells outgrowing the rectal crypt in a 2-day-old culture (×10).

**Figure 2:**
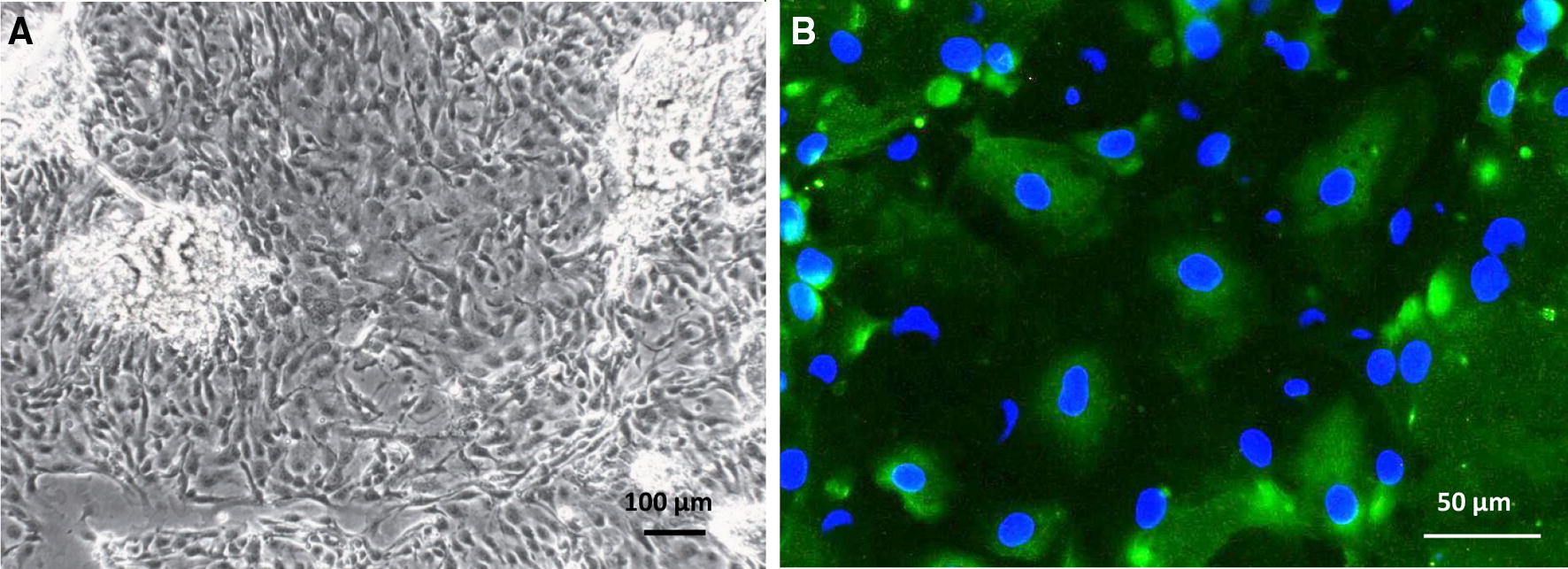
**Characterization of bovine rectal epithelial cells. A** The light microscopic image showing a development of a confluent monolayer of rectal epithelial cells after 6 days of culture (×10). **B** Characterization of rectal epithelial cells using a pan-cytokeratin specific FITC-labelled mouse monoclonal antibody (green) and Hoechst 33 258 nucleic acid stain (blue) (×40).

The suspension of single crypts (Figure 1A) was successfully obtained using collagenase (100 U/mL) in combination with mechanical disintegration (passing the crypt suspension through an injection needle) of rectal tissue. The crypts were seeded on collagen-coated slides. The collagen coating improved the attachment and subsequent growth and differentiation of the cells (data with and without the use of collagen not shown). Two to 3 days later, large epithelial cell clusters were present around the attached crypts (Figure 1B). A confluent monolayer was obtained at 6 to 7 days after seeding the crypts (Figure 2A). Short treatment with trypsin together with fetal calf serum deprivation was used to inhibit the growth of fibroblasts. The method was successful as confirmed by the staining of vimentin (data not shown), which is known as fibroblast intermediate filament. The vimentin-negative cells could be observed, thereby suggesting that only the epithelial cells were present in the culture. The epithelial character of the cells was further confirmed using the cytokeratin marker (Figure 2B).

### Proteomic analysis of bLF derived from milk

**Figure 3:**
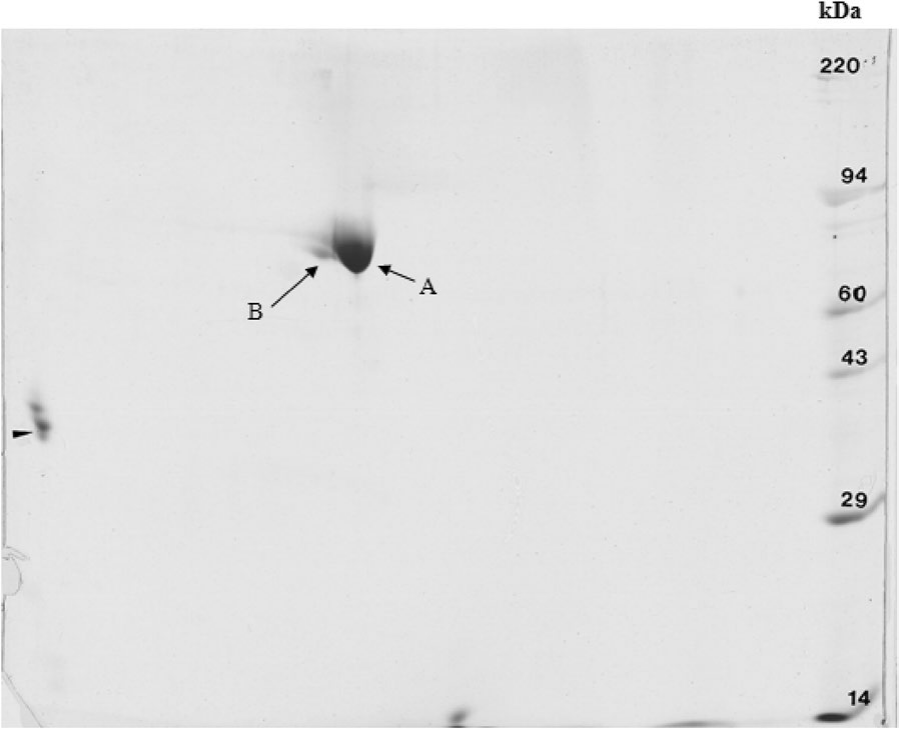
**2D gel electrophoresis of bLF purified from milk.** One μg of tropomyosin, giving a lower polypeptide spot of a molecular weight of 33 000, pI 5.2, was added to the samples as internal marker (short arrow). Sample A (right longer arrow) and B (left longer arrow), as the two protein spots used for LC–MS/MS.

Two dimensional-PAGE of bLF derived from milk revealed two protein spots, designated sample A (~80 kDa) and sample B (~73 kDa) (Figure 3). The spots were analyzed twice, once in a short gradient and once in a long gradient, in order to find differences in the amino acid sequence of these two proteins.

Only one protein was found in both samples, namely bovine lactoferrin (Genbank AAA30610). The short gradient revealed protein sequence coverages of 70 and 65% for sample A and B, respectively (Additional file 1). The long gradient revealed protein sequence coverages of 71 and 68% for sample A and B, respectively. To explain the differences in mass of these proteins, we looked for truncations or alternative splicing of the protein at the N- or C-termini. We found full sequence coverage for the C-terminus in both sample A and B (Additional file 1), so difference in the samples due to truncation or alternative splicing at the C-terminus of these proteins was excluded. Subsequently, we looked for differences at the N-terminus. Bioinformatics analysis of lactoferrin allowed to identify two lactoferrin isoforms in the human protein. One of them was the isoform with the canonical amino acid sequence called lactoferrin and the second one was the lactoferrin isoform called delta-lactoferrin, which is similar to lactoferrin, but it lacks the first 44 amino acids. Since the N-terminus also contains a sequence that has bactericidal and antifungal activity [35, 36], and it is present in lactoferrin but not in delta-lactoferrin, alternative splicing at the N-terminus was the second reason for which samples A and B could differ in molecular mass. However, in both samples, we found clear evidence that the N-terminus was fully intact (Additional file 1).

### Influence of EHEC O157:H7 on the intracellular localization of bLF

**Figure 4:**
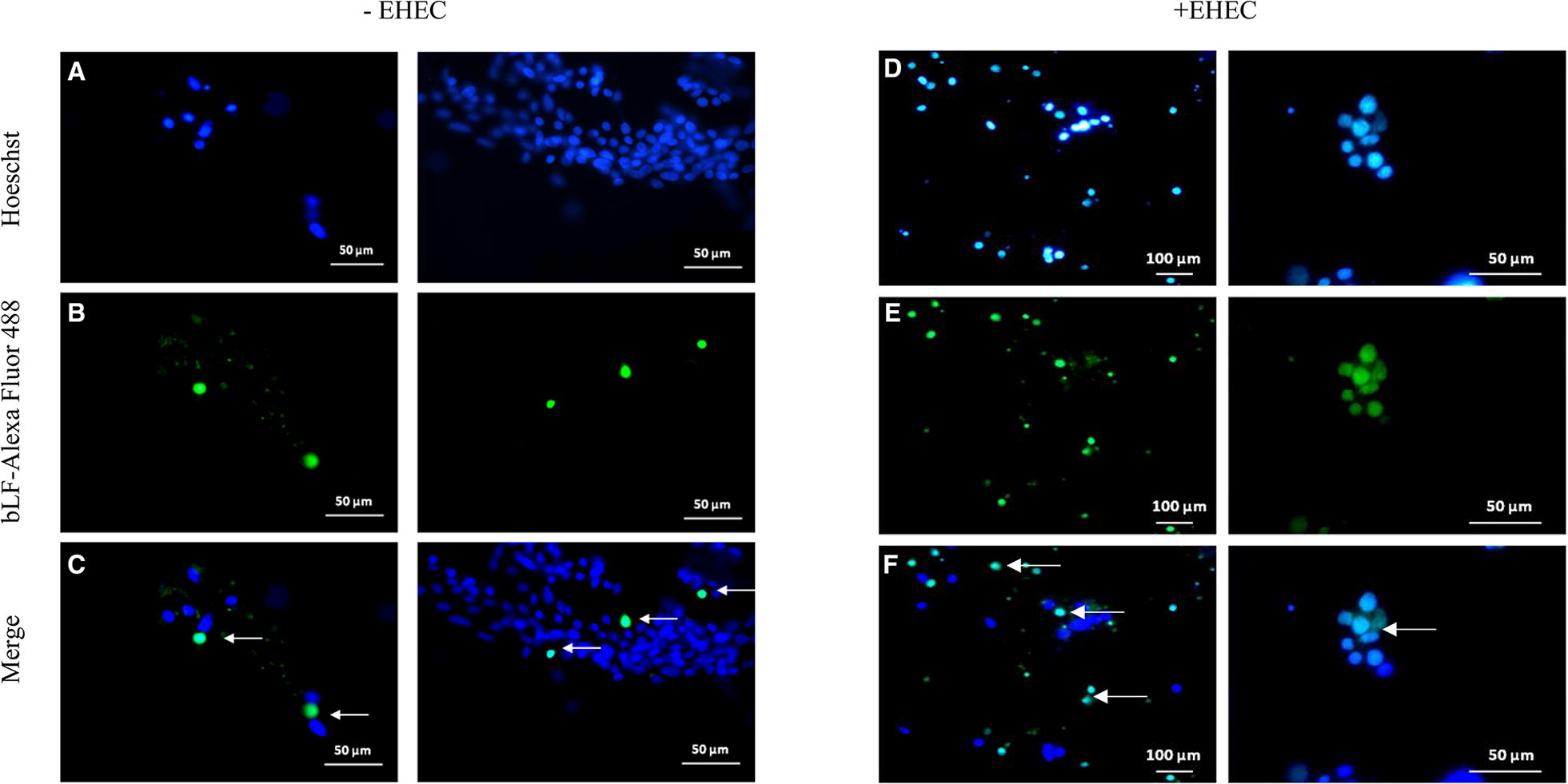
**Fluorescence microscopy of bovine rectal epithelial cells incubated with bLF-Alexa Fluor 488 (×40). A**, **D** shows stained cell nuclei. **B** shows the localization of bLF-Alexa Fluor 488 without EHEC infection. (**C**, **F**) Merged images showing bLF-Alexa Fluor 488 inside the nuclei (arrow). **E** shows the localization of bLF-Alexa Fluor 488 with EHEC infection.

**Figure 5:**
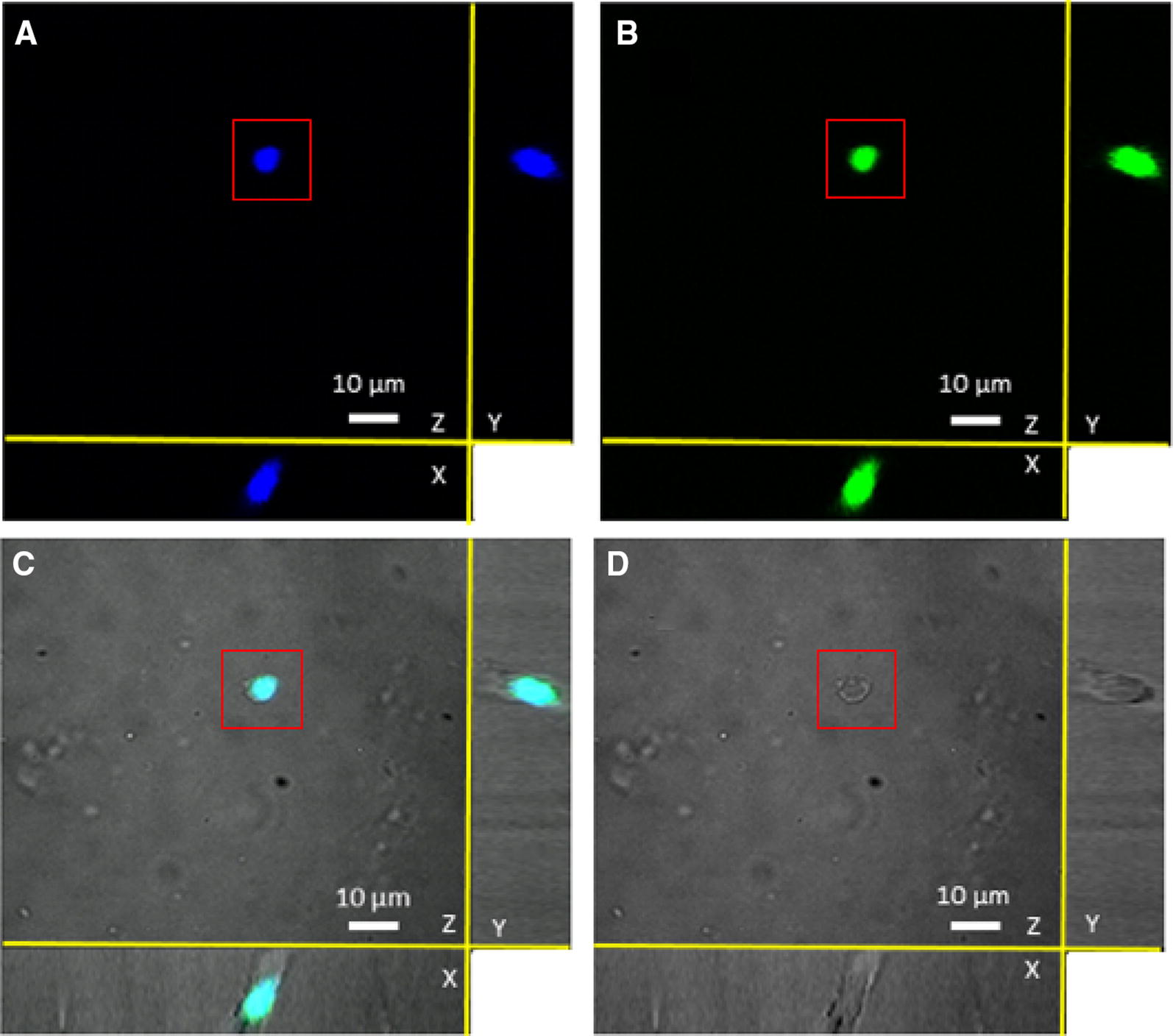
**Three-dimensional confocal identification of bLF internalized in the nucleus of a rectal epithelial cell.** The confocal identification was performed in the absence of EHEC. Three-dimensional reconstruction of the nucleus (**A**) and bLF (**B**). Merged 3D images (**C**) confirming presence of bLF in the nucleus. The corresponding cell, marked with red rectangle, that was chosen for 3D reconstruction (**D**).

**Figure 6:**
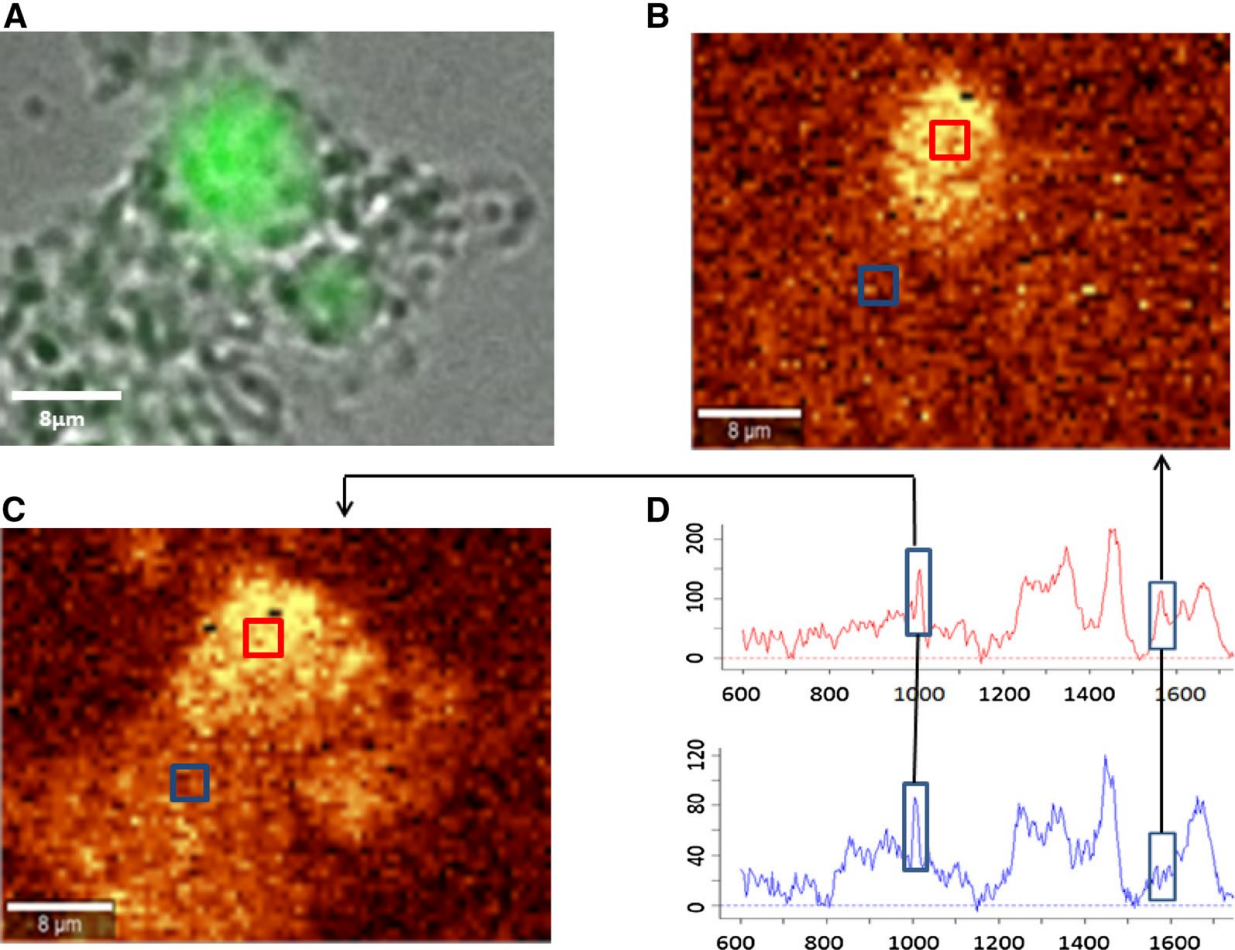
**Fluorescence and Raman images of a rectal epithelial cell containing bLF derived from milk.** Analysis from a cell in the absence of EHEC. **A** Fluorescence microscopy image of bLF-Alexa Fluor 488 in the cell. **B** The heat map generated using the 1555 cm^−1^ peak, which is highly intense in bLF. **C** The heat map generated using the 1003 cm^−1^ peak of phenylalanine, showing the protein distribution inside the cell. **D** Raman scattering spectra showing the peaks used for generation of the heat maps (**B**, **C**). The upper, red spectrum taken inside the nucleus, in the area marked with red rectangle on (**B**) and (**C**), contains the peak at 1555 cm^−1^. The bottom, blue spectrum, obtained outside the nucleus, in the area marked with blue rectangle on (**B**) and (**C**), has no 1555 cm^−1^ peak.

Bovine milk LF was internalized into bovine rectal epithelial cells and the protein translocated to the nucleus as demonstrated by confocal fluorescence microscopy (Figures 4A–C). The intranuclear localization of bLF could be confirmed by 3D-imaging (Figure 5; Additional file 2) and by Raman microscopy (Figure 6) using the spectrum of pure bLF in PBS as control (Additional file 3). The spectral peaks in the 1555 cm^−1^ (Figure 6D) were found to be common for two, random points inside the nucleus (marked with red rectangle) (Figures 6B and C) and for lactoferrin, confirming the nuclear localization of lactoferrin. This peak was not observed for the spectra of two random points outside of the nucleus (marked with blue rectangle) (Figures 6B and C). The control, Alexa Fluor 488 was never present in the nuclei of the cells.

Interestingly, nuclear translocation of bLF was significantly (*p* < 0.05) enhanced when the epithelial cells were inoculated with EHEC O157:H7. Fluorescence microscopy revealed the average of 17 on 1270 (1.34%) bLF-Alexa Fluor 488 positive nuclei in the absence of EHEC compared to the average of 480 on 1248 (38.5%) bLF-Alexa Fluor 488 positive nuclei in the presence of EHEC (Figures 4D–F).

## Discussion

### Isolation and characterization of bovine rectal epithelial cells

The recto-anal junction is the predominant colonization site of *E. coli* O157: H7 in cattle. Therefore, the primary cell culture was used for examining the intracellular localization of milk bLF in the presence and absence of EHEC O157:H7. As far as we know, only three studies have described the isolation of bovine epithelial cells from adult cattle [25–27] and they all mention the use of enzymatic disintegration of rectal tissue by collagenase (75 U/mL) in combination with accutase or dispase. Key features of the methods described so far and the method used in this study are summarized in Table 1. We could successfully obtain a suspension of single crypts using collagenase (100 U/mL) alone in combination with mechanical disintegration (passing the crypt suspension through an injection needle) of rectal tissue, similar to the study of Bridger et al. [37]. The use of only one enzyme enhanced the standardization and reproducibility of the experiment. The crypts were seeded on collagen-coated slides. The collagen coating improved the attachment and subsequent growth and differentiation of the cells (data with and without the use of collagen not shown). We used the same culture medium as Dziva et al., Mahajan et al. and Sheng et al., with the exception of the presence of 10% FCS instead of 2.5% FCS during the first 24 h [25–27]. Two to 3 days later, large epithelial cell clusters were present around the attached crypts. A confluent monolayer was obtained at 6 to 7 days after seeding the crypts, which was also observed by Sheng et al. [26], although they used a higher number of crypts (400 versus 250 crypts per 24-well in our study). We seeded a lower number of crypts to reduce the risk of fibroblast overgrow of our rectal epithelial cells. Fibroblast overgrowth is a major problem encountered during culture of more slowly growing specialized cell types. However, the use of lesser crypts as described by others, did not prevent fibroblast overgrow [26, 27]. Until now, several methods have been described for the reduction of fibroblast growth in primary intestinal epithelial cell cultures. These methods include slow serum (≤ 2%) supplementation of growth media [28], the use of l-valine deficient medium [29], short treatment of cultures with trypsin, dispase or pepsin [38], density gradients to enrich the crypt epithelium in the original cell suspension [39] and the use of *cis*-OH-proline [27]. In this study, a short treatment with trypsin together with fetal calf serum deprivation was used to inhibit the growth of fibroblasts. The method was successful as confirmed by the staining of vimentin (data not shown). The epithelial character of the cultured cells was successfully confirmed using the cytokeratin marker.

**Table 1:** **Key features of methods described to prepare primary epithelial cell cultures from rectal biopsies of adult cattle**

	Dziva et al. [25]^a^	Sheng et al. [27]	Present study
Enzymatic disintegration	Collagenase 75 U/mL Dispase 20 mg/mL	Collagenase 75 U/mL Accutase 20 mg/mL	Collagenase 100 U/mL
Mechanical disintegration	No	No	Yes
Differential centrifugation	Yes	Yes	Yes
Insulin	0.25 U/mL	0.25 U/mL	0.25 U/mL
Epidermal growth factor	10 ng/mL	10 ng/mL	10 ng/mL
FCS	2.5%	2.5%	10% during first 24 h Afterwards 1%
Number of crypts planted	400–600	400	200–250
Coating with collagen	Yes	Yes	Yes
Fibroblast growth inhibition	d-Valine	*Cis*-OH-proline	Repeated gentle trypsinization Reduction of FCS to 1%

^a^The method described by Mahajan et al. [26] was identical as the one used by Dziva et al. [25].

### Proteomic analysis of bLF derived from milk

Since the N- and C-termini of the bLF proteins found in sample A and B were found to be intact, there is no truncation, no alternative splicing on this protein. Therefore, sample A and B both contained secreted bLF as determined by LC-MS/MS. An isoform lacking the first 44 amino acids as described for ∆LF in humans, was absent in bovine milk. One of the reasons that ∆LF was not detected in bovine milk may be the intracellular expression of ∆LF in many human cells. It is a healthy tissue marker known to act as a transcription factor [21]. The only logical explanation for the difference in mass between the protein in sample A and B is either internal truncation, which is questionable but possible, or most probably non-glycosylation or de-glycosylation of the protein of 73 kDa. To find out, we could perform *N*-glycan, *O*-glycan and glycolipid analyses. Furthermore, additional differences in post-translational modifications between the proteins in sample A and B could also explain the difference in mass.

### Nuclear translocation of bLF and added value of confocal Raman microscopy

Human ∆LF clearly targets the cell nucleus by use of the nuclear localization signal (NLS) “RRSDTSLTWNSVKGKK” (position 417–432; C-lobe) and binds to *SKP1*, *BAX*, *DCPS* and *SELH* promotors, regulating cell proliferation or apoptosis depending on the target gene pathway [11, 17, 19, 20, 40]. Human LF possesses a pentapeptide “GRRRR” (position 1–5; N-lobe) which might act as nuclear localization signal sequence (NLS) [41]. As far as we know, the presence of a NLS has not been described for bLF.

Nuclear targeting of secreted LF is still controversial. Thus, it was interesting to notice that bLF derived from milk translocated to the nucleus of rectal epithelial cells as confirmed by 3D confocal fluorescence analysis and confocal Raman microscopy. The latter technique is of added value to this research. Fluorescence-based imaging techniques can perform analyses on small biosamples, such as the rectal epithelial cells used in this study, but the use of labels restrict the analytical value of the fluorescence images. Raman microscopy combines confocal imaging with Raman spectroscopy. Raman spectroscopy is a non-destructive, label-free technique used to determine the chemical composition of a sample [42–46]. The underlying principle of confocal Raman microscopy is based on light scattering. Thus, scattered light gives information about molecule’s vibrations, providing a Raman spectrum, with different bands corresponding to the vibrational frequencies of different functional groups. Each molecule therefore has a unique fingerprint, or spectrum, according to its chemical bonds. Raman microscopy can simultaneously detect the location and the amounts of multiple compounds such as proteins, lipids, DNA, and RNA [47]. Raman microscopy has been proven a useful technique in revealing conformational changes of proteins, being very sensitive both to changes in secondary structure as well as in the microenvironment of the side chains [48]. It has already been used to study the thermal stability of human lactoferrin over a wide pH range (4.0–9.0) [49]. In this study, the peak in the 1555 cm^−1^, that was highly intense for bLF, can be assigned to the Trp indole ring [49]. Trp residues are involved in protein folding forming both native and non-native hydrophobic contacts even in denatured proteins to ensure their proper folding [50]. Further studies revealed that peptides rich in Trp amino acids were found to possess the highest antimicrobial activities [51].

Bovine LF translocated to the nucleus of rectal epithelial cells. However, less than 2% of the rectal epithelial cells contained bLF in their nuclei. A similar finding was described by Suzuki et al. [52], studying the internalization of hLF by Caco-2 cells by confocal laser scanning microscopy. They found the intestinal LF receptor, known as intelectin [53, 54] together with hLF in the nucleus of a limited number of cells. In the case of Caco-2 cells, this was probably due to the fact that the intestinal LF receptor was only expressed in a subpopulation of Caco-2 cells [54]. Nielsen et al. [55], dissolved human lactoferrin in the medium of porcine small intestinal mucosal explants and detected hLF occasionally in the nucleus of T cells present in the lamina propria. Thus, most studies described the presence of LF inside nuclei of a limited number of human or porcine intestinal cells, which is in accordance with our observations for bovine rectal epithelial cells. However, Puddu et al. [56] found bLF in the nucleus of up to 100% freshly isolated human monocytes, which was correlated with IL-6 expression. However, their capacity to internalize bLF rapidly decreased during their differentiation into dendritic cells and the authors assumed, although the bLF entry mechanism in human monocytes is not known, that this could be due to differences in the expression of e.g. CD14 and/or DC-SIGN in monocytes versus monocyte-derived DCs.

### Influence of EHEC O157:H7 on the intracellular localization of bLF

The established method for the preparation of a primary bovine rectal epithelial cell culture was subsequently used for examining the intracellular localization of milk bLF in the presence and absence of EHEC O157:H7. The fact that lactoferrin is localized in the nucleus of the rectal epithelial cells was one of the most important findings of this study. Moreover, the percentage of cells having bLF in their nuclei significantly increased, when rectal epithelial cells were incubated with *E. coli* O157:H7. Nevertheless, further analysis is needed to understand the effect of bLF nuclear translocation on rectal epithelial cell function, and to investigate how *E. coli* O157:H7 enhances the nuclear translocation of bLF. EHEC colonisation perhaps augments the expression of bLF receptors, resulting in an increased percentage of cells having bLF in their nuclei. Elevated expression of bLF receptors could perhaps be the result of iron uptake by the bacteria, lowering the iron availability for the cell, whereafter the cell reacts with an elevated expression of bLF receptors. However, in that case, the expression of transferrin receptors is also expected to augment. The latter could be examined during future experiments. On the other hand, this phenomenon could be part of a more general innate defence mechanism of the cell, as lactoferrin is part of the innate immune system. This could be examined by repeating the study while inoculating the cells with another bacterial or even viral pathogen.

## Supplementary information

**Additional file 1. MASCOT search results.** Only one protein was found in both samples, namely bovine lactoferrin (Genbank AAA30610). The short gradient revealed protein sequence coverages of 70 and 65% for sample A and B, respectively. The long gradient revealed protein sequence coverages of 71 and 68% for sample A and B, respectively.

**Additional file 2. Three-dimensional confocal identification of bLF internalized in the nucleus of a rectal epithelial cell.** The confocal identification of bLF-Alexa Fluor 488 was performed in the absence of EHEC. Three-dimensional reconstruction of the nucleus (E) and bLF (F). Merged 3D images (G) confirming presence of bLF-Alexa Fluor 488 in the nucleus.

**Additional file 3. Control Raman scattering spectra.** The Raman scattering spectra for the Alexa Fluor 488 dye (green) and for unlabelled milk bLF (orange) are shown in the left and right panels, respectively. The 1555 cm^−1^ peak marked with the blue rectangle is highly intense in the bLF spectrum.

## Data Availability

All data generated or analysed during this study are included in this published article (and its additional files).
