# Supplementary figures and images for: Lactoferrin translocates to the nucleus of bovine rectal epithelial cells in the presence of Escherichia coli O157:H7
Source: Vet Res. 2019 Oct 1;50:75. doi: 10.1186/s13567-019-0694-3 (PMC6771091; doi:10.1186/s13567-019-0694-3)

**
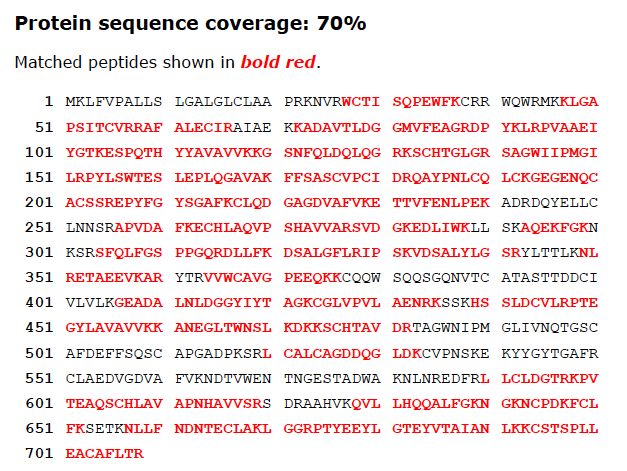
**

**
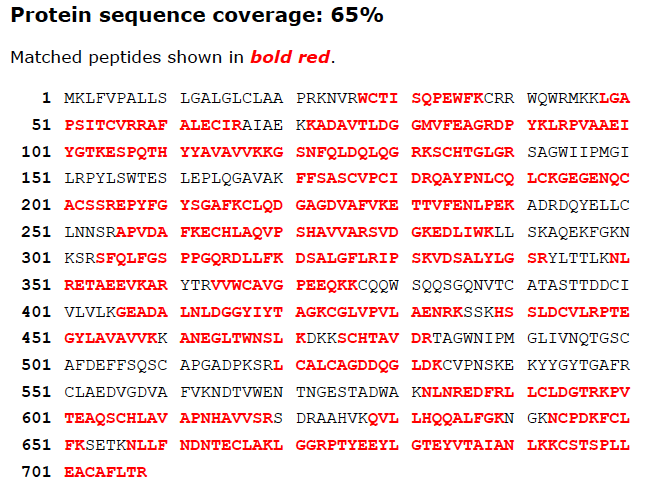
**

**
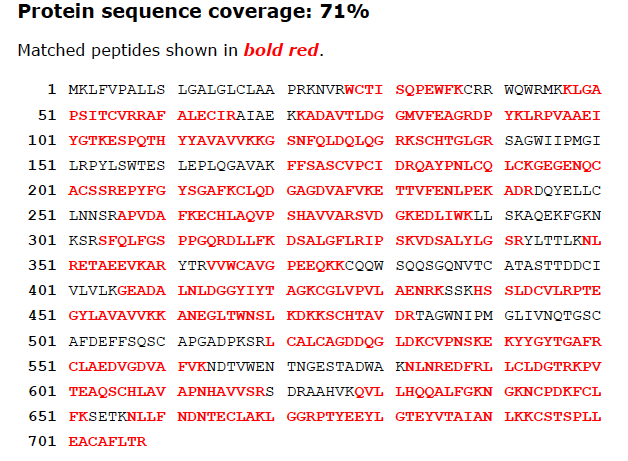
**

**
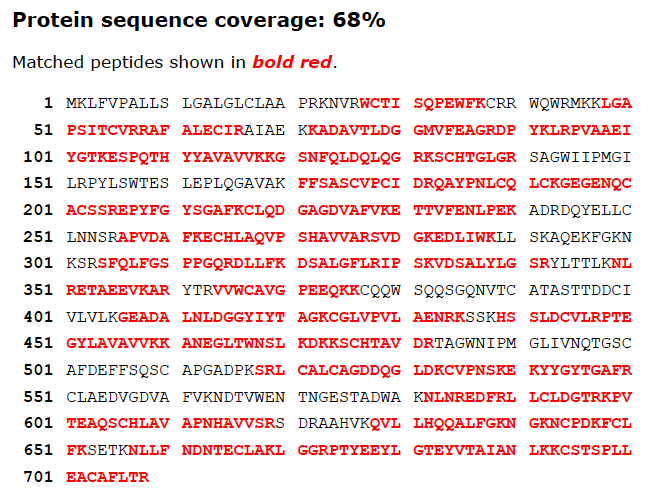
**

Supplement: Supplementary file 1 — Additional file 1. MASCOT search results. Only one protein was found in both samples, namely bovine lactoferrin (Genbank AAA30610). The short gradient revealed protein sequence coverages of 70 and 65% for sample A and B, respectively. The long gradient revealed protein sequence coverages of 71 and 68% for sample A and B, respectively. [file 13567_2019_694_MOESM1_ESM.docx]

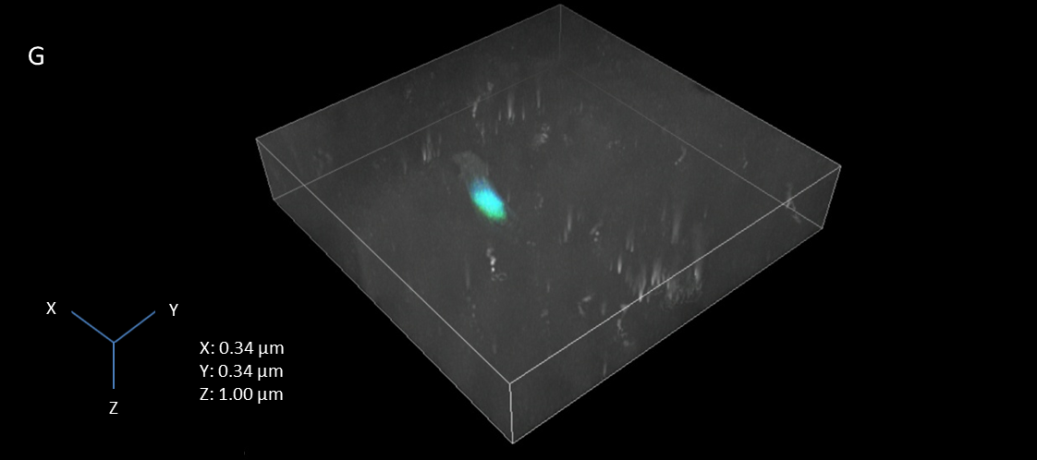

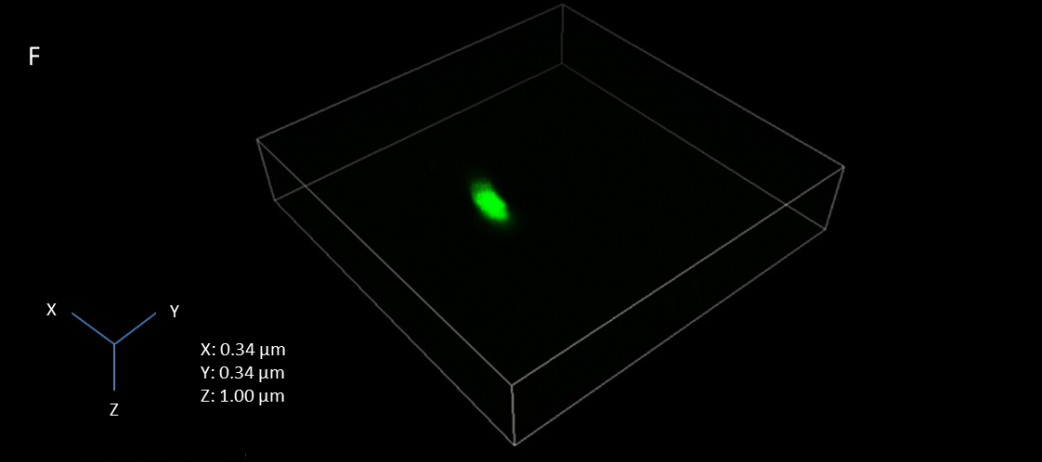

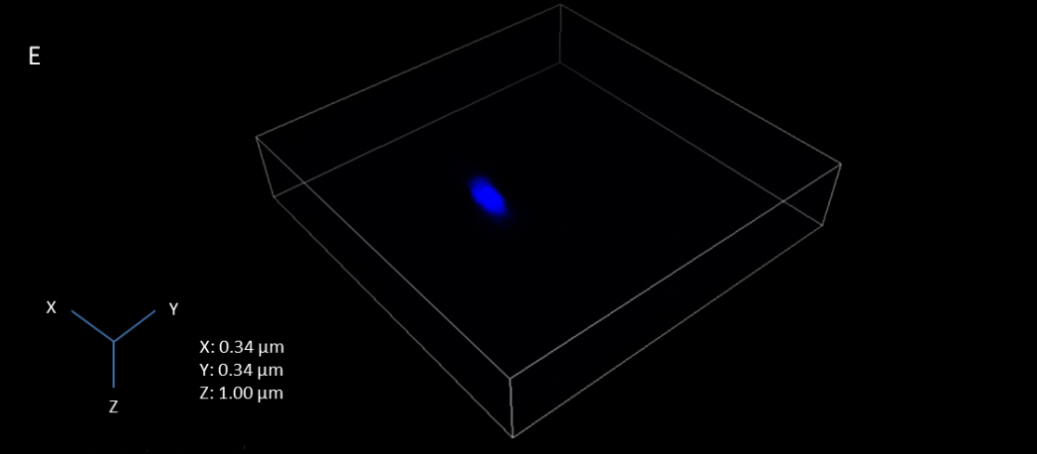

Supplement: Supplementary file 2 — Additional file 2. Three-dimensional confocal identification of bLF internalized in the nucleus of a rectal epithelial cell. The confocal identification of bLF-Alexa Fluor 488 was performed in the absence of EHEC. Three-dimensional reconstruction of the nucleus (E) and bLF (F). Merged 3D images (G) confirming presence of bLF-Alexa Fluor 488 in the nucleus. [file 13567_2019_694_MOESM2_ESM.docx]

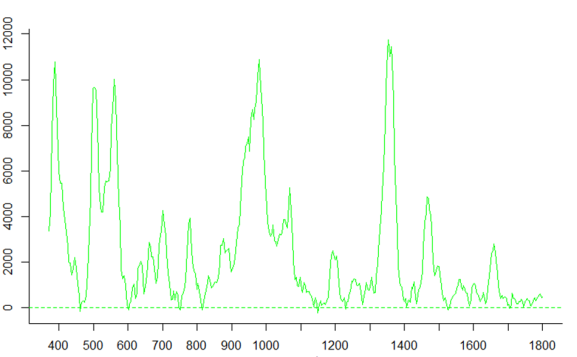

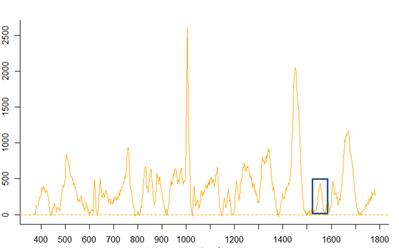

Supplement: Supplementary file 3 — Additional file 3. Control Raman scattering spectra. The Raman scattering spectra for the Alexa Fluor 488 dye (green) and for unlabelled milk bLF (orange) are shown in the left and right panels, respectively. The 1555 cm−1 peak marked with the blue rectangle is highly intense in the bLF spectrum. [file 13567_2019_694_MOESM3_ESM.docx]
